# Commentary: LACTB is a tumour suppressor that modulates lipid metabolism and cell state

**DOI:** 10.3389/fphys.2017.00396

**Published:** 2017-06-08

**Authors:** Ove Eriksson, Maciej Lalowski, Dan Lindholm

**Affiliations:** ^1^Biochemistry and Developmental Biology, Faculty of Medicine, University of HelsinkiHelsinki, Finland; ^2^Minerva Foundation Institute for Medical Research, Biomedicum HelsinkiHelsinki, Finland

**Keywords:** LACTB, mitochondria, phospholipids, lipid metabolism, cell proliferation, human cancer

Mitochondria are not only the power plants of the cell, producing ATP through oxidative phosphorylation, but are also involved in other essential processes such as intermediate metabolism, calcium signaling, and cell death. Findings in a recent paper by Keckesova et al. (2017), from the team of Robert Weinberg, provide compelling evidence that mitochondrial phospholipid metabolism is linked to cell proliferation and tumor formation. Specifically, the mitochondrial protein LACTB was found to influence the production of phosphatidylethanolamine (PE) from phosphatidylserine (PS) by the enzyme PS decarboxylase 1 (PISD1). This finding defines a role for LACTB in tumor suppression, acting through membrane lipid synthesis which may be useful for designing novel therapies targeting specific subtypes of cancer.

Mitochondria have evolved from alpha-proteobacteria, through a process called endosymbiosis. Many mitochondrial proteins are evolutionarily related to bacterial proteins although their functions may have diverged. LACTB is a mitochondrial intermembrane space protein present in all vertebrates derived from the bacterial penicillin-binding/β-lactamase protein family involved in peptidoglycan synthesis (Smith et al., 2001; Peitsaro et al., 2008). The amino acids essential for the catalytic activity of the penicillin-binding/β-lactamase proteins are fully conserved in LACTB, although mitochondria do not synthesize peptidoglycan. It is evident that LACTB must have been adopted for novel function(s) in the eukaryotic cell.

The current study reveals that LACTB exerts a tumor suppressor function by curbing mitochondrial phospholipid synthesis. The background for this ground-breaking discovery was the observation that LACTB is highly expressed in post-mitotic cells compared with dividing cells, suggesting a role in cell differentiation. In line with this, LACTB was downregulated in several neoplastic cells compared with non-tumorigenic cells, and furthermore, LACTB was reduced in 34–42% of breast cancers, whilst present in 100% of normal mammary glands. Introduction of LACTB into a panel of tumorigenic cells led to growth inhibition and expression of cell differentiation markers. In contrast, LACTB over-expression had little or no effect on growth or phenotype of non-tumorigenic cells. In solid tumors LACTB expression reduced the growth rate and promoted apoptosis, and sometimes led to a complete regression of the tumors, as observed in mice *in vivo*. In the context of expression of the *HRASG12V* or *MYCT58A* oncogenes, reduction in LACTB expression also induced tumor formation. These findings demonstrate that downregulation of LACTB can promote cell transformation, showing that LACTB has tumor-suppressive properties.

Using a broad range of techniques to assess mitochondrial function, the authors discovered that LACTB expression reduced the level of two central phospholipids, PE and lysoPE in tumorigenic cell lines. Supplementation of tumorigenic cells with lysoPE following LACTB expression preserved their tumorigenic phenotype, revealing a causal link between PE and LACTB. PE is synthesized from PS catalyzed by PISD1 (Di Bartolomeo et al., 2017), the genetic disruption of which is embryonically lethal (Steenbergen et al., 2005). LACTB over-expression induced a drop of 60–95% in the level of PISD1 with a concomitant reduction in PE. Targeting PISD1 by RNA interference had a similar effect as LACTB in the tumorigenic cell lines. This shows that LACTB acts through PISD1 although the mechanism remains open, as the authors did not observe any direct cleavage of PISD1 by LACTB. It is therefore possible that LACTB has other mitochondrial substrate(s) that directly or indirectly act on PISD1, or that LACTB influences PISD1 by some other mechanism.

PISD1 consists of two subunits, α and β, formed by the autocatalytic cleavage. The β subunit carrying the PISD1 activity is localized in the intermembrane space and the α subunit is attached to the inner mitochondrial membrane, whilst the substrate PS is present in the outer mitochondrial membrane (Aaltonen et al., 2016). LACTB is localized in the intermembrane space where it forms filaments spanning between the mitochondrial membranes (Polianskyte et al., 2009). Therefore, it is possible that LACTB can influence the accessibility of PS to PISD1 by controlling the spatial distance between the membranes, offsetting the effects of the mitochondrial contact sites and cristae organizing system (MICOS) (Aaltonen et al., 2016) (see Figure 1). This model is in line with the observation that PISD1 is not a direct substrate for LACTB, but that its activity is locally restrained by the presence of LACTB. Such mechanism may at least partially explain why LACTB had little effect when expressed in non-tumorigenic or post-mitotic cells.

**Figure 1:**
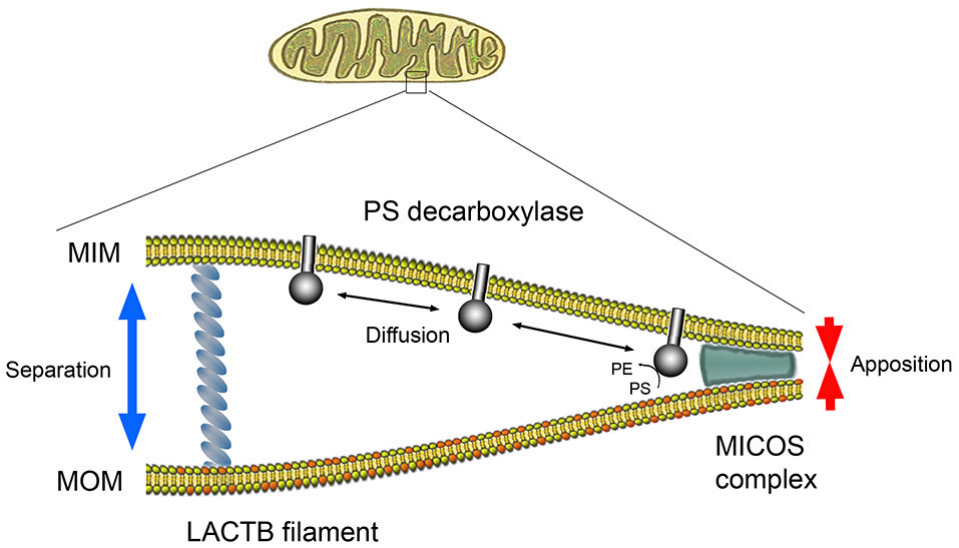
Hypothetical model of PS decarboxylase activity regulated by the spatial arrangement of the outer and inner mitochondrial membranes. Conversion of substrate PS (red) into PE in the outer mitochondrial membrane (MOM) is carried out by PISD1 protruding from the inner mitochondrial membrane (MIM). For PS decarboxylase to convert PS to PE, the MOM and MIM need to be in close apposition determined by the mitochondrial contact sites and the cristae organizing system (MICOS). LACTB may influence the degree of membrane apposition and hence the rate of PE formation.

Intriguingly, the authors report that LACTB carrying the R496K mutation, found in the MCF7-RAS breast cancer cells, was devoid of tumor-suppressive effect and lacked capability to inhibit PE synthesis when introduced into tumorigenic cells. This substitution mutation is located close to the C-terminus of LACTB and did not affect the catalytic activity of LACTB as assessed by a test peptide. It is of interest that the LACTB^R496K^ variant resulting from a single nucleotide substitution (A**G**G->A**A**G), normally occurs in the human population with an allele frequency of 71% globally, but with large variations between different geographic regions. Future studies may reveal whether individuals homozygous for the R496K mutation are at increased tumor risk.

Previous studies have suggested a role for LACTB in obesity (Chen et al., 2008) and mitochondrial membrane organization (Polianskyte et al., 2009). The current study does not only provide crucial insight into LACTB function, but has also more far-reaching implications. Firstly, rapidly growing tumor cells require not only an abundance of nucleotides for DNA synthesis, but are also dependent on the availability of phospholipids for membrane biogenesis. Limiting the availability of membrane lipids could hence be an efficient mechanism to keep dividing cells on the leash. Secondly, major obstacles in the design of anticancer drugs include undesired off-target effects observed with many drugs due to similar ligand binding sites in different proteins. Consequently, unique proteins without paralogs in humans could provide more selective drug targeting opportunities (Rawlings, 2007). Pharmacological modulation of LACTB function could also prove useful for targeting alterations in metabolic disorders due to the link to obesity. The current study is an important step forwards, raising pertinent questions that will undoubtedly drive further studies.

## Author contributions

All authors listed have made substantial, direct and intellectual contributions to the work, and approved it for publication.

### Conflict of interest statement

The authors declare that the research was conducted in the absence of any commercial or financial relationships that could be construed as a potential conflict of interest.
